# Supplementary material for: Reconciling Mining with the Conservation of Cave Biodiversity: A Quantitative Baseline to Help Establish Conservation Priorities
Source: PLoS One. 2016 Dec 20;11(12):e0168348. doi: 10.1371/journal.pone.0168348 (PMC5173368; doi:10.1371/journal.pone.0168348)
Supplement: S1 Dataset — (ZIP) [file pone.0168348.s002.zip › Taxa/Serra Norte/SN_2013/N5S/Lista N5S-06.pdf]

CAVIDADE N5S-0006

Classe	Ordem	Fam/Outros	Gên/Outros	Espécie	S-U	U-S	Zon.
Arachnida	Acari	Asternoseiidae		sp.84	X		E
Arachnida	Acari	Ixodidae		jovem	X		E
Arachnida	Acari	Mesostigmata		jovem	X		E
Arachnida	Amblypygi	Phrynidae	*Heterophrynus*	*longicornis*	1	X	E
Arachnida	Araneae	Araneidae	*Alpaida*	aff. *negro*	X		E
Arachnida	Araneae	Barychelidae		jovem	1		E
Arachnida	Araneae	Ctenidae		jovem	2	1	E
Arachnida	Araneae	Drymusidae		jovem	X		E
Arachnida	Araneae	Filistatidae		jovem	X		E
Arachnida	Araneae	Mysmenidae	*Microdipoena*	sp.1	X		E
Arachnida	Araneae	Ochyroceratidae	*Speocera*	sp.1	X	X	E
Arachnida	Araneae	Oonopidae	Oonopinae	jovem		X	
Arachnida	Araneae	Pholcidae	*Mesabolivar*	aff. *cambridgei*	X		E
Arachnida	Araneae	Pholcidae	*Mesabolivar*	sp.1	X		E
Arachnida	Araneae	Pholcidae		jovem	X	X	E
Arachnida	Araneae	Salticidae		jovem	X		E
Arachnida	Araneae	Tetragnathidae		jovem	X		E
Arachnida	Araneae	Theraphosidae		jovem	1		E
Arachnida	Araneae	Theridiidae	*Theridion*	sp.1	X	X	E
Arachnida	Araneae	Theridiosomatidae	*Plato*	sp.1	X		E
Arachnida	Araneae	Uloboridae	*Uloborus*	sp.	X	X	
Arachnida	Pseudoscorpiones	Bochicidae		sp.1	X		E
Arachnida	Pseudoscorpiones	Chernetidae	*Spelaeochernes*	sp.1	X	X	E
Arachnida	Pseudoscorpiones	Chthoniidae		jovem	X		E
Chilopoda	Scutigeromorpha	Pselliodidae	*Sphendononema*	*guildingii*	1	X	E
Diplopoda	Polydesmida	Pyrgodesmidae		jovem		X	
Diplopoda	Siphonophorida	Siphonophoridae		jovem	X		E
Diplopoda	Spirostreptida	Pseudonannolenidae	*Pseudonannolene*	sp.1	1	X	
Entognatha	Collembola	Cyphoderidae		sp.1	X		E
Entognatha	Collembola	Entomobryidae		sp.9	X		E
Entognatha	Collembola	Paronellidae		sp.4	X		E
Insecta	Blattodea	Polyphagidae		sp.1	4	X	E
Insecta	Coleoptera	Chrysomelidae		sp.5	X		E
Insecta	Coleoptera	Chrysomelidae		sp.7	X		E
Insecta	Coleoptera	Elateridae		sp.1	X		E
Insecta	Coleoptera	Rhipiphoridae		sp.1	X		E
Insecta	Diptera	Chironomidae		sp.	X		E
Insecta	Diptera	Dolichopodidae		sp.	X		E
Insecta	Diptera	Keroplatidae	*Euceroplatus*	sp.	X		E
Insecta	Diptera	Micropezidae		sp.	X		E
Insecta	Diptera	Psychodidae	Phlebotominae	sp.	X		E
Insecta	Diptera	Tabanidae		sp.	X		E
Insecta	Heteroptera	Dipsocoroidea		sp.	X		E
Insecta	Heteroptera	Lygaeidae		jovem	X		E
Insecta	Heteroptera	Reduviidae	Reduviinae	sp.	1	X	E
Insecta	Hymenoptera	Chalcidoidea		sp.		X	
Insecta	Hymenoptera	Formicidae	*Camponotus*	sp.1	X	X	E
Insecta	Hymenoptera	Formicidae	*Camponotus*	sp.2	X		E
Insecta	Hymenoptera	Formicidae	*Eurhopalothrix*	sp.1	X		E

Insecta	Hymenoptera	Formicidae	*Pachycondyla*	sp.2	X		E
Insecta	Hymenoptera	Formicidae	*Paratrechina*	sp.2	X		E
Insecta	Hymenoptera	Formicidae	*Pheidole*	sp.2	X		E
Insecta	Isoptera	Termitidae	*Nasutitermes*	sp.	X	X	
Insecta	Lepidoptera	Galacticidae		sp.	X		E
Insecta	Lepidoptera	Gelechioidea		sp.	X		E
Insecta	Lepidoptera	Noctuoidea		sp.5	1		E
Insecta	Lepidoptera	Noctuoidea		sp.7	1		E
Insecta	Lepidoptera	Noctuoidea		sp.8	2		E
Insecta	Lepidoptera	Tineoidea		jovem		X	
Insecta	Neuroptera	Myrmeleontidae		jovem	X		E
Insecta	Orthoptera	Gryllidae		sp.	1		E
Insecta	Orthoptera	Phalangopsidae	*Paraclodes*	sp.	9	X	E
Insecta	Orthoptera	Phalangopsidae	*Phalangopsis*	sp.	3		E
Insecta	Psocoptera	Epipsocidae	*Epipsocus*	sp.2	X		E
Insecta	Psocoptera	Manicapsocidae	*Nothoentomum*	sp.1	X		E
Insecta	Psocoptera	Ptiloneuridae	*Triplocania*	sp.9	X		E
Insecta	Psocoptera			jovem	X	X	E
Insecta	Thysanura	Nicoletiidae		sp.1	X		E
Gastropoda	Pulmonata	Systrophiidae	*Happia*	sp.	X		E
Amphibia	Anura	Strabomantidae	*Pristimantis*	cf. *fenestratus*		3	E
Mammalia	Chiroptera	Emballonuridae	*Peropteryx*	sp.		X	
Mammalia	Rodentia	Echimyidae	*Proechimys*	sp.		1	E
